# An Integrated System of Multifaceted Machine Learning Models to Predict If and When Hospital-Acquired Pressure Injuries (Bedsores) Occur

**DOI:** 10.3390/ijerph20010828

**Published:** 2023-01-01

**Authors:** Odai Y. Dweekat, Sarah S. Lam, Lindsay McGrath

**Affiliations:** 1 Department of Systems Science and Industrial Engineering, Binghamton University, Binghamton, NY 13902, USA; 2 Wound Ostomy Continence Nursing, ChristianaCare Health System, Newark, DE 19718, USA

**Keywords:** cost-sensitive support vector machine, genetic algorithm, hospital-acquired pressure injuries, predictive model, pressure ulcer, bedsores, integrated system, pressure injuries

## Abstract

Hospital-Acquired Pressure Injury (HAPI), known as bedsore or decubitus ulcer, is one of the most common health conditions in the United States. Machine learning has been used to predict HAPI. This is insufficient information for the clinical team because knowing who would develop HAPI in the future does not help differentiate the severity of those predicted cases. This research develops an integrated system of multifaceted machine learning models to predict if and when HAPI occurs. Phase 1 integrates Genetic Algorithm with Cost-Sensitive Support Vector Machine (GA-CS-SVM) to handle the high imbalance HAPI dataset to predict if patients will develop HAPI. Phase 2 adopts Grid Search with SVM (GS-SVM) to predict when HAPI will occur for at-risk patients. This helps to prioritize who is at the highest risk and when that risk will be highest. The performance of the developed models is compared with state-of-the-art models in the literature. GA-CS-SVM achieved the best Area Under the Curve (AUC) (75.79 ± 0.58) and G-mean (75.73 ± 0.59), while GS-SVM achieved the best AUC (75.06) and G-mean (75.06). The research outcomes will help prioritize at-risk patients, allocate targeted resources and aid with better medical staff planning to provide intervention to those patients.

## 1. Introduction

Hospital-Acquired Pressure Injuries (HAPIs) is one of the most common health conditions in the US, which costs more than $ 26.8 billion annually [1]. Known by many names, such as pressure injury (PI), bedsore, or decubitus ulcer, the injury develops due to pressure or pressure in combination with shear that results in tissue deformation or tissue ischemia. HAPI refers to these injuries that occur while admitted to the healthcare system [2].

**Figure 1:**
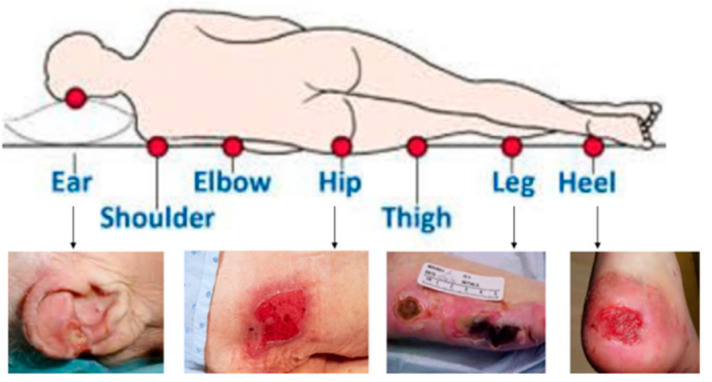
Different locations of HAPI [8].

HAPI can happen almost everywhere on the body; however, they seem to happen more frequently over bony prominences or behind medical equipment, as shown in Figure 1. Pressure injuries are staged according to the level of exposed tissue [2]. Stage 1 pressure injuries present as intact skin with a localized area of non-blanchable erythema. Stage 2 is partial thickness skin loss with exposed dermis or a serum-filled blister. Stage 3 is full-thickness skin loss in which adipose tissue is visible. Stage 4 is full-thickness skin loss and tissue loss which exposes underlying structures such as fascia, muscle, tendon, or bone [2]. Unstageable injuries include injuries where the extent of skin and tissue loss is obscured by slough or eschar. In contrast, a Deep Tissue Pressure Injury (DTPI) is a localized area of non-blanchable deep red, maroon, or purple discoloration, which may evolve rapidly as the extent of the injury is revealed [2].

When HAPI develops in the hospital, the patient’s length-of-stay increases, and the patient requires additional resources. From the perspective of the individual patient and family, there is a decreased trust in the health care system, which can lead to difficulty managing the patient’s care. The staff who care for the patient as well as the hospital system may face litigation and quality of care concerns from local, state, and national agencies [3].

Standardized risk assessment and targeted prevention treatments can prevent most HAPI cases [2]. However, most acute or long-term care patients are at risk based on the standardized risk assessment. Therefore, the expense of prevention in terms of labor and preventive goods can be high. The Braden scale is an example of a risk assessment tool that nurses use to identify patients at risk for early HAPI development and implement individualized preventative measures against pressure injuries [4,5,6,7].

In the last decade, researchers have utilized Machine Learning (ML) approaches to predict if patients would develop HAPI before it occurs by utilizing patients’ Electronic Health Record (EHR) and therefore reduce the HAPI rate. Until now, no studies have answered the research question of when HAPI occurs for at-risk patents. This research is the first to have an integrated system of multifaceted ML models to predict if and when HAPI occurs by collecting a new piece of information not introduced in the literature, i.e., time for HAPI. Furthermore, it is the first research that combines Genetic Algorithm (GA), Cost-Sensitive (CS) learning, and Grid Search (GS) with ML algorithms to provide an indication as to not only who will develop HAPI but also when HAPI is likely to occur by training and testing two integrated models for highly unbalanced problems.

This paper is structured as follows: Section 2 summarizes and criticizes the related literature in predicting HAPI. Section 3 describes the variables, data source, participants, and model development. Section 4 summarizes the results of the developed method and compares it with the most used classification algorithms. Section 5 discusses the output of the suggested results, their implications for the new approach in the medical field, and limitations of this research. Lastly, Section 6 provides the conclusion and future direction of this research.

## 2. Related Literature

A systematic literature review is applied to the field of applying ML in predicting HAPI. The database used in this criterion were PubMed, Web of Science, Scopus, and Science Direct. The timeline is between 2007 through July 2022. The database search keywords were pressure injury, pressure ulcer, machine learning, deep learning, data mining, hospital-acquired pressure injury, HAPI, early detection, predictive modeling of pressure injury, bedsores, decubitus ulcer, and others. In the end, 26 studies met the criteria. [9] is excluded because it was a survey on predicting HAPI using ML. The remaining 25 studies used ML methods to predict HAPI early [1,10,11,12,13,14,15,16,17,18,19,20,21,22,23,24,25,26,27,28,29,30,31,32,33].

HAPIs rate is considered a rare event in hospitals because it occurs among a minor volume of the total population [2]. Two studies out of 25 had a highly unbalanced dataset of less than 3% [20,26]. However, most of the other studies designed their samples to have a high portion of HAPI. For example, HAPI rate was 52.31% [10], 50.00% [16], 31.92% [18], 28.78% [19], 28.10% [22], 20.10% [25], and 20.00% [34].

Random Oversampling (RO) techniques were applied to most studies (i.e., in 76% of the studies) [1,10,11,14,15,16,18,19,20,21,22,23,25,26,28,30,31,32]. On the other hand, four studies used Synthetic Minority Oversampling Technique (SMOTE) to deal with the unbalanced dataset [12,13,24,27]. Oversampling techniques can be a solution by replicating the patients with HAPI (minor class); good results might be generated for training purposes. However, in implementation, overfitting might occur. Therefore, the algorithm will misclassify any HAPI cases and consider them as non-HAPI because it is already trained on a minor sample. Therefore, there is a need to use other advanced/hybrid models to overcome the challenge of the unbalanced dataset.

All studies used traditional ML methods to predict HAPI. Most of the researchers tried multiple approaches to the same study. The most used approaches are Logistic Regression (LR), which was used 17 times in the above studies during model development and experimentation [1,10,12,13,15,17,18,19,20,21,22,24,26,28,30,32,33], Random Forest (RF) was used 12 times [1,11,12,13,17,19,24,26,27,28,33,34], Decision Tree (DT) was used 10 times [1,14,15,18,19,20,24,25,30,33], Support Vector Machine (SVM) was used nine times [1,15,17,24,25,26,28,29,30], Multilayer Perceptron (MLP) was used eight times [1,12,17,24,25,27,28,29], and k-nearest neighbor (kNN) was used three times [19,27,28]. Other algorithms were used in the experimentations: Linear Discriminant Analysis (LDA) [27] and Adaptive Boosting (AdaBoost) [12].

Nevertheless, metaheuristic optimization was not used till now in this field to optimize the ML hyperparameters. Only two researchers used GS optimization [17,26]. Besides, CS learning was used only once to deal with the unbalanced HAPI dataset [17] and was applied to a benchmark dataset.

All researchers utilized ML algorithms to predict only which patients will develop HAPI before it occurs by utilizing the patient’s historical data in the EHR [1,10,11,12,13,14,15,16,17,18,19,20,21,22,23,24,25,26,27,28,29,30,31,32,33]. However, none of the studies predict when HAPI might occur for the predicted HAPI cases. Furthermore, the status of patients with HAPI changes during their stay in the hospital. All the studies used a snapshot of static data (i.e., the status of patients at admission or the most recent diagnosis of patients) [1,10,11,12,13,14,15,16,17,18,19,20,21,22,23,24,25,26,27,28,29,30,31,32,33]. However, no studies use variables at multiple points (i.e., multiple tests per patient, which change over time). However, having one record per patient does not count the changes in the status of patients during their stay.

In summary, no more than 25 studies have been conducted in this field that answer the question who will develop HAPI among the patients. This is insufficient and incomplete information for the clinical team because knowing who would develop HAPI in the future (classification tasks) does not help differentiate the severity and urgency of those predicted cases. Further, conducting prevention actions for all predicted patients would require more resources, time, and costs. On the other hand, none of the studies used metaheuristic and CS learning to deal with the highly unbalanced problem of predicting HAPI. Most of the studies used traditional ML approaches and oversampling techniques. Finally, all studies used one snapshot of the patient records (i.e., features) to predict HAPI. None of the above research considered the changes in patients’ status during their hospital stay.

This research will fill the above gaps in the literature by developing an integrated system of multifaceted ML models to predict if and when HAPI occurs. This model considers the changes in the status of patients at multiple points in time. It integrates GA with CS learning to enhance the performance of SVM when dealing with highly unbalanced HAPI dataset. This research is the first to fill the above gaps and address HAPI time in the literature to prioritize who is at the highest risk and when the highest risk will occur.

## 3. Research Methodology

### 3.1. Methods

The scope of this study is patients who were admitted to ChristianaCare hospital located in Delaware, USA and discharged between May 2020 and February 2022 (*n* = 15,889). Patients under 18 years old were excluded from the scope of this study. Additionally, labor and delivery patients, and emergency visits were excluded. Patients with HAPIs (*n* = 485) were identified through nurse documentation of validated HAPIs.

### 3.2. Data Source

The variables were extracted from a SQL database that pulls information from patients’ EHR. Wound, Ostomy, Continence (WOC) nurses kept track of patient records with validated HAPIs by manually documenting their notes in tracking documents; that was the source for identifying HAPI patients (*n* = 485). These documents included admission date and time, HAP occurrence date and time, and multiple variables across different points of time during the patient visit. The final dataset included the unique record per patient visit represented in Figure S1 in Supplementary Materials.

### 3.3. Variables

The goal of this study is to predict patients’ risk for developing HAPI and when they are expected to develop HAPI based on multiple risk factors. Ninety-eight risk factors that include the Braden risk assessment subscales were used as inputs for a ML model as summarized in Table S1 in supplementary material. These variables were selected based on the previous literature survey and clinicians’ feedback. Changing variables were measured based on three points of time across the patient stay; on admission (First), before discharge (Last), and the average value of all measured values of variables during stay.

This model had only 485 patients who had HAPI; therefore, predicting HAPI time did not provide accurate results. Therefore, the problem was converted from predicting continuous time values to a classification problem by labeling the time to develop HAPI into two categories; high-risk patients who are expected to develop HAPI in fewer than seven days, and medium-risk patients who are expected to develop HAPI in more than seven days. The medical team confirmed this categorization of risk where seven days is an adequate period to provide earlier preventive actions. Furthermore, the clinical team will use the output of predicting HAPI time to stratify at-risk patients. Therefore, even if a continuous prediction of the time is found, the predicted time will be converted into risk satisfaction: at high risk to provide an immediate intervention and at medium risk to provide later interventions.

### 3.4. Model Development

**Figure 2:**
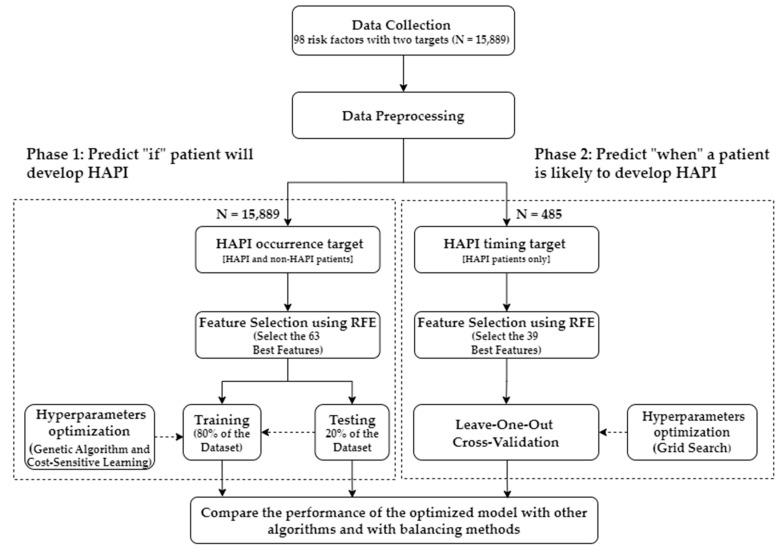
The framework of the proposed approach.

The integrated system of multifaceted ML models has been developed in two separate phases. Phase 1 predicts if the patient will develop HAPI, and Phase 2 predicts when HAPI is expected to occur for at-risk patients as shown in Figure 2. Having two different models allows for flexibility in selecting the most important features for each phase, one target per phase, which uses different optimized parameters and validation processes. However, the data preprocessing step was the same for both phases. The patients’ distribution in each phase is summarized in Table 1.

Preprocessing data before developing a ML model is an important step to ‘maximize’ information and knowledge extraction from variables. It also reshapes the data into an appropriate format that is readable by ML algorithms. Imputation, normalization, and categorization are all examples of preprocessing applied to the dataset to remove errors and missing values.

Feature selection was applied to select the top important variables for predicting HAPIs. Recursive Feature Elimination (RFE) was the algorithm used to perform that; this algorithm fits a predictive model to predict class through iterations of removing variables from weakest to strongest until an optimal set of variables is selected [35,36].

#### 3.4.1. Phase 1: Predict “If” Patient Will Develop HAPI

Phase 1 integrates GA with CS-SVM (GA-CS-SVM) to predict if a patient will develop HAPI before occurrence. This model considers 485 HAPI and 15,404 non-HAPI patients (*n* = 15,889); each patient has 98 risk factors/features. RFE was used to select the best features that affect HAPI development. The top 63 features were selected and used to develop the SVM model. The dataset was divided into 80% for training the model with 10-fold cross-validation, and the remaining 20% was used to test the model’s performance. GA was used to fine-tune the CS learning parameters to deal with the highly unbalanced training dataset and to enhance the performance. In this case, GA was used to select the best combination of class weights for False Negative (FN) and False Positive (FP) as explained below. Two oversampling techniques (SMOTE and RO) [37] were applied and compared for the same dataset to validate the suggested approach in terms of sensitivity, AUC, Geometric Mean (G-mean), and False Positive Rate (FPR). Furthermore, the performance of the optimized SVM (i.e., GA-CS-SVM) was compared to the non-optimized SVM to measure the optimization effect. Moreover, the seven commonly used algorithms in predicting HAPI in the literature were applied and tested to validate the suggested SVM model. A *t*-test compared the performance between GA-CS-SVM vs. non-optimized SVM. In contrast, Analysis of variance (ANOVA) was used to determine if there are any statistical differences between the means of all other validation methods, which includes GA-CS-SVM. Lastly, a *t*-test was used to measure the difference between the optimized SVM and the best method among other methods. The confidence level is kept at a 0.05 error margin for 50 experiments.

SVM is a supervised ML algorithm that is heavily used in the medical field. Research shows that it provides decent classification results in predicting HAPI. Nine out of 25 studies that predict HAPI adopted SVM [1,15,17,24,25,26,28,29,30]. SVM can deal with simple and complex classifications and is robust to high-dimensional data [38]. This research deals with 63 features selected by FRE and adopts a linear SVM. 

**Figure 3:**
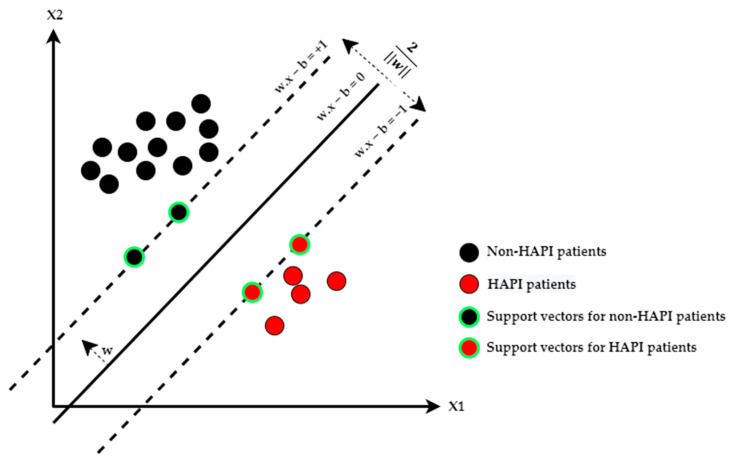
SVM algorithm.

The objective of SVM in binary classification problems is to find a margin hyperplane that maximizes the minimum distance to the hyperplane (i.e., find the optimal separation line with the maximum margin w between data points of two classes) [38], as shown in Figure 3. Having the optimal separation line means solving the mathematical optimization model below [39,40].

(1) $$min\;\frac{1}{2}\left|\left|w\right|\right|{\;}^{2}+C\;\sum_{1}^{N}\xi_{i}\;$$

(2) $$y_{i}\left(w^{T}x+b\right)\geq 1-\xi_{i}\;i=1,\;\ldots ,\;N$$

(3) $$\xi_{i}\geq 0\;i=1,\;\ldots ,\;N$$

whereas x is the HAPI dataset with N patients, x = ($x_{1},\;x_{2},\;\ldots ,\;X_{N}$), $x_{i}$, i = 1, …, N represents a patient with m features, and $y_{i}$ ∈ {0,1}, 0 denotes non-HAPI patients, and 1 denotes patients with HAPI. w represents the separating margin, C is the penalty parameter or regularization parameter that balances the margin w and the training error (i.e., loss). $\xi_{i}$ is the slack variable penalty during training, i = 1, ..., N, ξ ∈ $R_{+}^{N}$, and b is the bias or scalar offset [39,40].

The drawback of the above SVM structure is that it is inefficient to deal with highly unbalanced datasets. It is because C is fixed, and all data points (patients) are treated as equally likely during the training process. Therefore, the Cost-Sensitive SVM (CS-SVM) or Biased Penalties SVM (BP-SVM) [38,39] introduces different penalty coefficients $C_{1}$ and $C_{0}$ for HAPI and non-HAPI SVM slack variables during the training process [39,40]. The formulation for CS-SVM is provided below:

(4) $${\;}_{w,b,\xi }^{\mathrm{argmin}}\frac{1}{2}\;\left|\left|w\right|\right|{\;}^{2}+C\left[C_{1}\underset{\left\{i|y_{i}=1\right\}}{\sum }\xi_{i}+C_{0}\underset{\left\{i|y_{i}=0\right\}}{\sum }\xi_{i}\right]$$

(5) $$y_{i}\left(w^{T}x+b\right)\geq 1-\xi_{i}\;i=1,\;\ldots ,\;N$$

(6) $$\xi_{i}\geq 0\;i=1,\;\ldots ,\;N$$

where $C_{1}\;$represents the penalty/cost of a false negative (FN) that classifies HAPI as non-HAPI (i.e., the penalty for misclassification of HAPI cases or cost of minority class HAPI Type 2 error), and $C_{0}$ represents the penalty/cost of a false positive (FP) that classifies non-HAPI as HAPI (i.e., cost of majority class non-HAPI); therefore, CS-SVM assigns different costs/weights for FN, and FP, in most cases, $C_{1}$ > $C_{0}$. As a result, FN will be minimized because its cost will be high (more penalty for misclassification). Therefore, the algorithm will learn to avoid misclassifying HAPI records during the training process, which increases the model sensitivity.

The size of the search space has an infinite number of scenarios because $C_{1}$ and $C_{0}$ can be any positive real numbers (i.e., $C_{1}$ ∈ $R_{+}^{N}$, and $C_{0}$ ∈ $R_{+}^{N}$). To reduce the search space $C_{1}$ and $C_{0}$ are bounded between 1 and 100, which is deemed sufficient penalty to impose on the CS-SVM. Furthermore, it is assumed $C_{1}$ > $C_{0}$ as in the literature [39,40] . However, the search space is still infinite 0 < $C_{1}$ ≤ 100, 0 < $C_{0}$ ≤ 100, and $C_{1}$ > $C_{0}$, $C_{1}$, $C_{0}$ can be any real value within the boundaries. Therefore, there is a need for a heuristic method to find the semi-optimal solutions rather than trial and error in this infinite search space. 

There are several methods used in the literature to find the values of $C_{1}\;\mathrm{and}\;C_{0}$ in CS-SVM, such as Kernel-based Possibilistic c-Means (KPCM) algorithm, random search, intuition, Fuzzy SVM (FSVM), Bayes consistent classier, 2ν approach, incremental CS learning, self-adaptive cost weights-based CS Large margin Distribution Machine (CS-LDM) [39,40,41,42,43,44,45,46,47,48,49].

GA is an evolutionary algorithm that uses a stochastic approach for global search. It has been used heavily to optimize hyperparameters for general ML methods and SVM [40,50,51,52,53,54,55,56,57,58]. GA is used in this research as a robust heuristic tool to identify the semi-optimal distribution of $C_{1}$ and $C_{0}$ that satisfies Equations (4)–(6). CS-SVM is used as an emulator that represents the GA’s objective function, which maximizes the AUC. The AUC of CS-SVM is calculated based on a 10-fold cross-validation of the training set.

The pseudocode of the hybrid GA-CS-SVM is shown in Algorithm 1. This algorithm optimizes the CS-SVM parameters, which are encoded as real values in GA; initially a set of random solutions is selected, and each solution gets included in the training process. Once training is complete, each solution is validated with the 10-fold cross-validation, which represents the fitness value of each solution.
**Algorithm 1. Combining GA-CS-SVM for optimizing the CS learning parameters**1: Set GA parameters (Pc, Pm, n, gmax) $2:\;\mathrm{Encode}\;\mathrm{solutions}\;(\mathrm{CS}\;\mathrm{learning}\;\mathrm{parameters}:C_{1}$, $C_{0}$) using real value encoding 3: Randomly generate n solutions 4: Calculate the fitness value (AUC) of each solution by the trained CS-SVMs 5: **for** i = 1 to gmax **do** 6:       **for** j = 1 to n/2 **do** 7:             Select two parents 8:             Crossover to create two children with Pc 9:              Mutate children with Pm 10:       **end for** 11: Replace parents with children 12: **end for** 13: Return the best solution

For every GA generation, a tournament selection process runs over k solutions to select two parents for breeding. The selected pair are combined using the crossover operator and then mutated to create two mutated children (solutions). The process of selection, crossover, and mutation is iterated until a certain number of solutions/children is generated. After that, the selected children replace an entire generation for the next iteration. The solutions keep evolving until the predefined maximum number of generations (gmax) is reached. Then, the best solution is delivered through the hybrid GA-CS-SVM that has the highest AUC value.

For the GA, the following parameters were used: tournament selection to select the parents (k = 2), population size = 50, 100 generations as a stopping criterion, crossover probability (Pc) and mutation probability (Pm) were 1.00 and 0.01, respectively. A weighted average of 60% of parent 1 and 40% of parent 2 was used to combine the two parent solutions. A Gaussian distribution was used with mean 0, and standard deviation of 0.01 to find the mutation value. Finally, the optimal values of $C_{1}$ and$\;C_{0}$ to achieve the best AUC were 43.32 and 6.26, respectively.

#### 3.4.2. Phase 2: Predict “When” a Patient Is Likely to Develop HAPI

Phase 2 combines GS with SVM (GS-SVM) to predict the second target, which is the timing of HAPI for at-risk patients. This model considers only the 485 patients with HAPI, with 98 risk factors. One hundred thirty-five patients developed HAPIs within the first seven days (at high risk), and the remaining developed HAPI after seven days (at medium risk). The distribution for the high-risk vs. medium-risk patients is 28.00% vs. 72.00%.

RFE was utilized to select the top features that impact the HAPI timing. Therefore, the 39 best features were selected and used as inputs in Phase 2. Leave-one-out Cross-Validation (LOOCV) was adopted in Phase 2 to deal with the small HAPI dataset [59]. Therefore, the learning algorithm was applied once for each record, which uses all other records as a training set and the selected patient as a single-patient test set. Because the dataset is not highly unbalanced as in Phase 1, there was no need to use CS learning. Instead, a GS with a 10-fold cross-validation was used to tune and select the best hyperparameters of the SVM.

Alternatively, SMOTE and RO were used with LOOCV. The performance of the optimized SVM (i.e., GS-SVM) was compared to the non-optimized SVM. In addition, the seven algorithms used in Phase 1 were used to validate the suggested model in Phase 2. Unlike the 80% training vs. 20% testing, LOOCV was applied once for each patient. Therefore, there is no variability in such a method. Therefore, confidence levels are not available for the results of Phase 2.

In GS, the algorithm searches exhaustively through a predefined manual subset of the hyperparameter space of SVM [60]. The hyperparameters used for the GS are the following: kernel type to be used in the SVM [Linear, Polynomial, Radial Basis Function, Sigmoid], the regularization parameter C [1, 10, 100, 1000], gamma for Radial Basis Function [0.0001, 0.001, 0.1, 1], and polynomial degree [2, 3, 4]. The algorithm provides the best combination of hyperparameters with the best performance when the regularization parameter C is 1, the kernel is linear, and gamma is 1.

### 3.5. Performance Metrics

Check for overfitting was conducted through comparison of the model’s performance metrics on training and testing sets; this included sensitivity, G-mean, AUC, and False Positive Rate (FPR). Refer to Table 2 for detailed explanation of the confusion matrix metrics that include False Negative (FN), False Positive (FP), True Negative (TN), and True Positive (TP). FN represents HAPI patients who were missed by the model. FP represents healthy patient who were predicted by model to have HAPI. TN represents healthy patients predicted correctly by the model. TP represents HAPI patients who were correctly predicted as at risk by the model.

Sensitivity is the ratio of TP to all actual cases with HAPIs. FPR measures the probability of non-HAPI cases predicted as HAPI cases. G-Mean measures the balance between classification performances on both the majority non-HAPI and minority HAPI cases. Lastly, AUC measures the ability of the model to distinguish between patients with and without HAPI [61].

(7) $$\mathrm{Sensitivity}=\frac{\mathrm{TP}}{\mathrm{TP}+\mathrm{FN}}\times 100\%$$

(8) $$\mathrm{FPR}=\frac{\mathrm{FP}}{\mathrm{FP}+\mathrm{TN}}\times 100\%$$

(9) $$G-\mathrm{mean}=\sqrt{\left(\frac{\mathrm{TP}}{\mathrm{TP}+\mathrm{FN}}\right)\times \left(\frac{\mathrm{TN}}{\mathrm{TN}+\mathrm{FP}}\right)\times 100\%}$$

## 4. Results

This research developed an integrated system of multifaceted ML models to predict if and when HAPI occurs for at-risk patients. This study collected data for 15,889 patients with a 3% HAPI rate. Ninety-eight risk factors with two targets for each patient were collected and preprocessed. The two targets are HAPI occurrence and time to develop HAPI from admission to the occurrence. Risk factors that indicate the patient’s status were collected at three points. These factors change due to the status of the patients during the length-of-stay. Comprehensive features and diagnoses were collected to represent patients’ environment, such as demographical factors, lab factors, medical device factors, medications, diagnosis factors, and medical factors. Two different types of predictive models were investigated in two phases. Phase 1 integrated GA as a heuristic method with CS-SVM (GA-CS-SVM) to handle the high imbalance HAPI dataset to predict the occurrence of HAPI. RFE was used in Phase 1 to extract the best features. The dataset was separated into training with 10-fold cross-validation and testing. Phase 2 adopted LOOCV to train a different model to predict when HAPI will occur for HAPI patients. In this phase, the 39 best features were selected by RFE. GS was used to optimize the hyperparameters of the SVM in Phase 2. Both phases were compared to the seven algorithms used to predict HAPIs. Moreover, two oversampling methods were compared to the proposed approach. Statistical tests were performed to highlight the statistical significance of the proposed approach. Lastly, for each phase, Random Forest was used to measure each feature’s influence on prediction.

**Figure 4:**
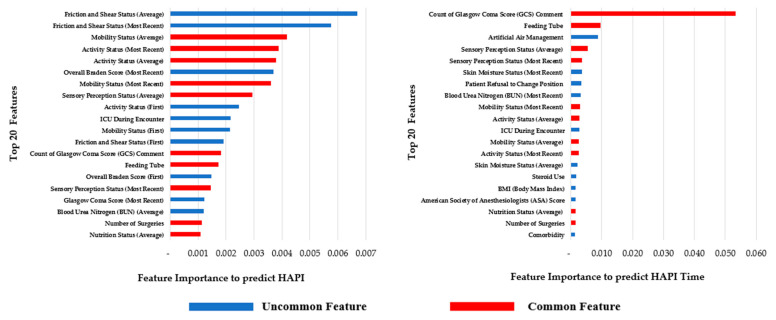
Top 20 features for phase 1 and phase 2.

Figure 4 shows the top 20 features for each phase; the red-labeled features are common top features such as Count of Glasgow Score (GCS) comment, Feeding Tube, Number of Surgeries, and some Braden subscales such as Sensory Perception Status (Average), and Mobility Status (Average). Having two phases with a feature selection for each phase’s target allows flexibility in selecting the most important features.

In Phase 1 (predicting if HAPI will happen), GA-CS-SVM achieved the best sensitivity (74.29), AUC (75.79), and G-mean (75.73) compared to the most common algorithms used in predicting HAPIs (SVM, LR, AdaBoost, LDA, KNN, DT, RF, MLP) and when compared to other oversampling methods (RO and SMOTE). The results are summarized in Table 3. Training with 10-fold cross-validation and testing results were presented to reflect no overfitting for the proposed method. It is worth mentioning that overfitting happened when adopting SMOTE. A 95% Confidence Level (CL) of 50 experiments was implemented for each scenario.

**Figure 5:**
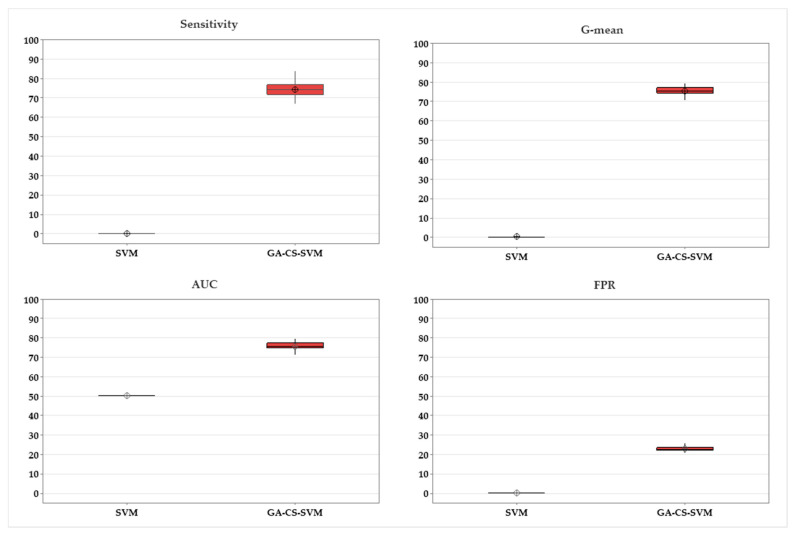
Comparison between GA-CS-SVM and SVM.

*t*-test shows that there is a statistical significance between (un-optimized) SVM and an optimized one (GA-CS-SVM) in terms of sensitivity, AUC, G-mean, and FPR (*p*-value < 0.05) as shown in Table 4 and presented in Figure 5.

**Figure 6:**
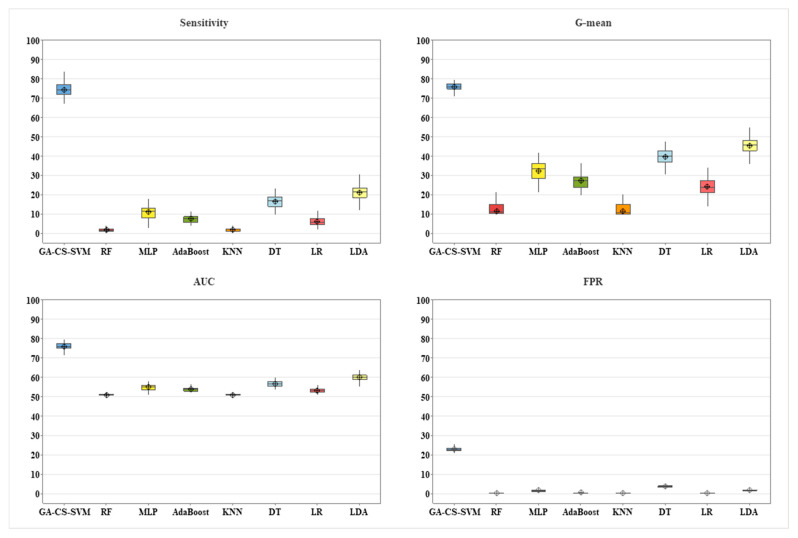
Results for GA-CS-SVM vs. other methods in terms of performance metrics (phase 1).

**Figure 7:**
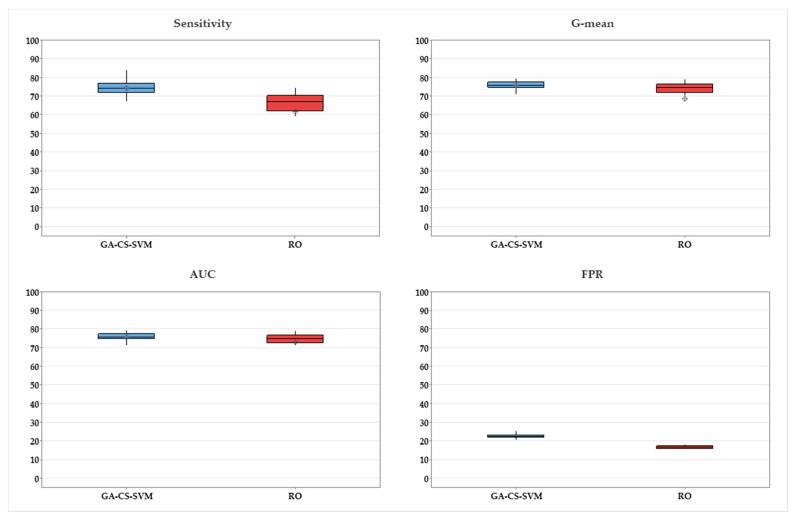
Comparison between GA-CS-SVM and balancing using RO.

ANOVA is performed to determine a significant difference between all algorithms by testing for differences in means using variance, as shown in Table 4. *p*-value is less than 0.05 for sensitivity, AUC, G-mean, and FPR, which indicates a statistical difference among the methods. Figure 6 presents the mean and confidence interval for GA-CS-SVM vs. other methods in terms of sensitivity, AUC, G-mean, and FPR. It is observed that GA-CS-SVM performs the best. However, oversampling technique has acceptable results compared to the remaining techniques. Therefore, the *t*-test is performed between GA-CS-SVM and balancing using oversampling. Table 4 shows a statistical significance between RO and GA-CS-SVM in terms of sensitivity, AUC, G-mean, and FPR (*p*-value < 0.05), as presented in Figure 7.

**Figure 8:**
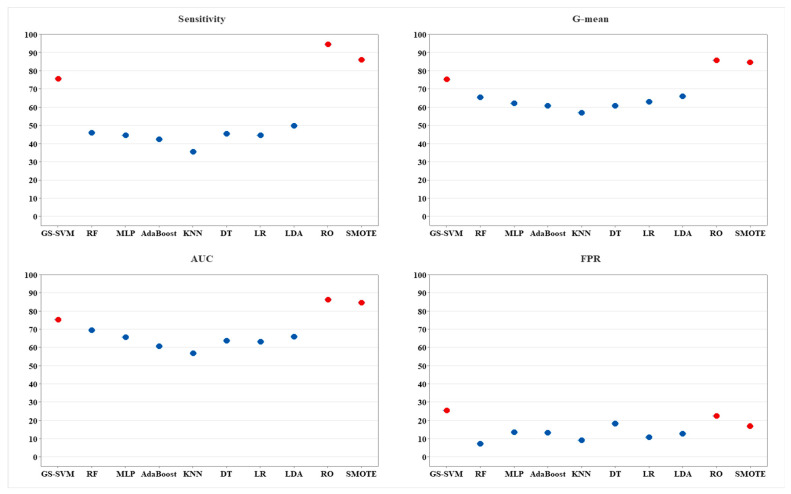
Results for GS-SVM vs. other methods in terms of performance metrics (phase 2).

In Phase 2 (predicting when HAPI happens), the optimized SVM (i.e., GS-SVM) achieved the best sensitivity (75.56), AUC (75.06), and G-mean (75.06) compared to the most common algorithms, as shown in Figure 8. However, balancing methods with LOOCV (RO and SMOTE) perform better than the GS-SVM because they had similar samples from the training set, which increases the chances of overfitting. In contrast, GS-SVM adopted LOOCV without oversampling. Therefore, the results are less than the oversampled one. Table 5 summarizes the performance metrics for GS-SVM and all other techniques. As discussed in the methodology section, the LOOCV is applied once for each patient, using all other patients as a training set and the selected patient as a single-patient test set. Therefore, there is no variability and statistical tests as applied in Phase 1. However, it is observed that the effect of optimization for SVM that uses the GS technique on SVM (GS-SVM) increases the sensitivity from 59.26 to 75.56 for instance, whereas AUC increased from 72.49 to 75.06.

## 5. Discussion

Phase 1 adopted 63 features using RFE; Phase 2 identified 39 features using RFE; 30 are common for both phases, as shown in bold in Table S1 in Supplementary Materials. Most of the Braden Scale subfactors are common factors, which further validates that the Braden Scale is a critical assessment. The proposed approach takes the daily assessment to a new level, one that would not feasibly be able to be performed by the nurse who cares for the patient; it can take a combination of the first score, average score, and most recent score when determining HAPI and the timing of HAPI. In addition to the Braden Score provided by nurses who care for the patient throughout the hospitalization, the proposed model takes all other historical factors into account, such as the prior year’s inpatient visit count, the number of surgeries, specific comorbidities; all factors that help determine the level of risk but are things that would require significant additional resources if performed by the nurse who cares for the patient at the bedside.

**Figure 9:**
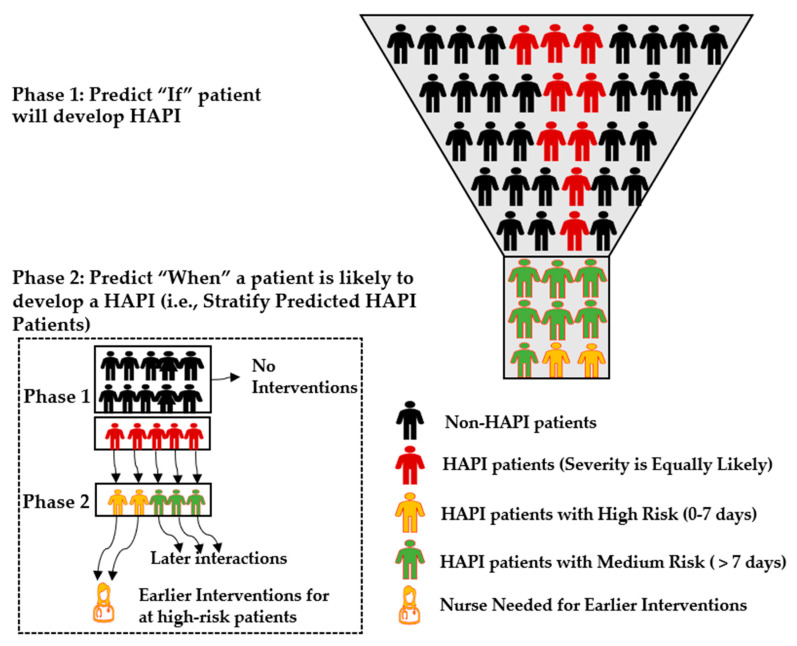
Implications of the proposed research on the medical team.

At the basis of the Orlando theory of nursing process is the holistic assessment of the patient, formulation of nursing diagnosis, planning, implementation, and evaluation of plan of care [62]. Assessment tools have been developed to assist with the identification of at-risk patients for some of the most common nursing diagnoses, that include patients at risk for development of HAPIs. The currently available risk assessment scores have adequate specificity and sensitivity to identify patients at risk but have an elevated false positive rate to identify a large number of at-risk patients who do not develop pressure injuries. Phase 1 of the ML project predicted which patients will develop HAPIs, the second phase is aimed at prioritizing at-risk patients by predicting the timing of HAPI. The clinical implications of not only knowing who is at risk of developing HAPI, but also when during their hospitalization this is likely to occur are many. The first and easiest to measure is the reduction in HAPIs, which results in reduced length-of-stay and reduced financial penalties. The second is a reduction of patient harm, which results in reduced financial penalties and increased external hospital ratings/confidence of care. For the bedside staff, the ability to target and focus on those patients most at risk for development of HAPIs will result in better patient care and appropriate allocation of resources. Using ML in combination with clinical assessment tools, there is a reduction in the number of patients identified as being at risk; the addition of the likely time frame of development will further reduce the number of patients who require advanced prevention techniques. Targeting highest-risk patients will allow for nursing and support staff to individualize the care plan and allocate the greatest resources to those most at risk, while continuing to provide appropriate nursing care to all at-risk patients as shown in Figure 9.

Phase 1 helped to narrow down which patients are at most risk; the second phase helps determine the time frame the patient is most likely to develop a pressure injury during the hospital stay. According to the Agency for Healthcare Research and Quality (AHRQ), the average hospital length-of-stay was 4.5 days in 2021; however, the length-of-stay for vulnerable populations and those with chronic conditions tends to be longer. The average length-of stay for a person who experiences homelessness is around 6.5 days [63], whereas those in recent years who experience COVID-19 complications can have an average length-of-stay > 25 days [64]. Socioeconomic factors can also increase the risk of prolonged length-of-stay in the hospital; a small percentage of hospitalized patients have a length-of-stay measured in months [65]. The second phase of the project further stratifies level of risk over time, which helps to prioritize not only who is at risk, but when they will be at highest risk. Phase 2 gives patients’ level of risk per week. The time frame of seven days was chosen secondary to guidance from Kottner et al. (2019) [2] on the development of a DTPI. Development of a DTPI takes place up to 72 h prior to visibility at skin level [2]. Minimal intervention in a DTI would be 72 h, but an earlier intervention would better maximize interventions and skin condition; however, beginning intense interventions too early can be burdensome for the patient and caregiver. Seven days of intervention will help maximize tissue tolerance and reduce the risk of prolonged pressure over bony prominences, which is the goal in pressure injury prevention [2].

All of the proposed models in the literature were offline models that did not run on active patients’ records and update their inputs continuously, and because these models where built and tested on static dataset that did not change across patient stays, static data represent a summary snapshot of the data for the patient stay regardless of how many days were spent in the hospital [1,10,11,12,13,14,15,16,17,18,19,20,21,22,23,24,25,26,27,28,29,30,31,32,33], In contrast, this research considers the changes of patients’ records at three points of time [66].

The study methods used to gather the information for the HAPI risk score uses a combination of subjective and objective information gathered from the patient’s chart. The Braden Scale performed by the bedside nurse has an inter-rater reliability of 87.1% for overall score but tends to be lower at the level of the Braden Risk subscale level, for example, of moisture inter rater reliability of only 13% [67]. Differences among nurses who perform the Braden Scale may provide inaccurate or an incomplete picture of the patient’s current level of functioning. Furthermore, the suggested approach does not consider all the changes in patients’ status from admission to discharge; it considers the first, last, and average status during the length-of-stay. 

## 6. Conclusions and Future Work

HAPIs is one of the most prevalent health disorders in the United States, with yearly expenses exceeding $ 26.8 billion. It is often referred to as a pressure injury, bedsore, or decubitus ulcer. Sustained skin pressure can cause injuries to the skin’s underlying tissue. Most of the HAPI cases can be prevented through prevention interventions. Prevention is the most effective method for managing HAPIs. Most patients in acute care or long-term care settings are considered at risk, and the cost of interventions and prevention for all patients in terms of nurses and preventive products can be significant. In the literature, researchers utilized ML approaches to predict if patients would develop HAPI before it occurs by utilizing EHR to provide prevention actions. However, no more than 25 studies have been conducted in this field that answer the question who will develop HAPI among the patients. This is insufficient and incomplete information for the clinical team because knowing who would develop HAPI in the future (classification tasks) does not help differentiate the severity and urgency of those predicted cases. Further, conducting prevention actions for all predicted patients would require more resources, time, and costs. Moreover, patients predicted as at risk, most likely will remain at risk until discharge. Therefore, this research introduces for the first time a robust integrated ML approach to answer the question of not only who will develop HAPI, but also the timing of HAPI when it occurs for at-risk patients. The performance of the developed models is compared with state-of-the-art models in the literature, which achieved higher sensitivity, AUC, G-mean, and FPR than the eight most common algorithms used to predict HAPI and other balancing methods.

This research outcome will help the medical team prioritize the at-risk patients and allocate additional targeted resources to the patients who will likely have HAPIs during a specific time period (i.e., highest risk patients). Furthermore, this work will reduce patient harm (HAPI rate), which potentially reduces the length-of-stay. Lastly, it will help with better planning of medical staff to provide intervention to predicted HAPI patients and save costs, time, and resources. Future work will investigate the feasibility to automate the categories of the Braden Score to use a multidisciplinary approach to determine level of risk, which includes data pulled from Occupational or Physical Therapies, Registered Dietitian, as well as attending or consulting providers. Moreover, studies will utilize the online learning concept to capture all patients’ records during their stay (i.e., dynamic model). Lastly, a multi-task learning model can be developed for future work by training one dataset for both targets rather than training two different models separately.

## Figures and Tables

**Table 1:** Dataset distribution in phases 1 and 2.

**Phase 1** **(*n* = 15,889)**	**Target**	**If HAPI Developed or Not**	
	**Distribution of Phase 1**	Non-HAPI	HAPI
	**Number of Patients**	15,404 (97%)	485 (3%)
**Phase 2** **(*n* = 485)**	**Target**	**When HAPI developed for patients with HAPI**	
	**Distribution of Phase 2**	0–7 days (High-Risk)	>7 days (Medium Risk)
	**Number of Patients**	136 patients (28%)	349 patients (72%)

**Table 2:** Confusion matrix.

		**Predicted HAPI**	
		**Non-HAPI (0)**	**HAPI (1)**
**Actual HAPI**	**Non-HAPI (0)**	TN	FP
	**HAPI (1)**	FN	TP

**Table 3:** Phase 1 results (training with 10-fold cross-validation and testing).

Models			80% Training (10-Fold Cross-Validation)				20% Testing			
			Sensitivity	AUC	G-mean	FPR	Sensitivity	AUC	G-Mean	FPR
Proposed Approach	GA-CS-SVM	Mean	**74.06**	**75.67**	**75.65**	**22.71**	**74.29**	**75.79**	**75.73**	**22.71**
		* CL	**0.45**	**0.20**	**0.21**	**0.15**	**1.23**	**0.58**	**0.59**	**0.37**
Other Algorithms	SVM	Mean	0.01	50.00	0.10	0.01	0.01	50.00	0.10	0.01
		CL	0.01	0.00	0.20	0.00	0.01	0.00	0.20	0.00
	LR	Mean	6.55	53.19	25.45	0.16	5.87	52.86	23.83	0.15
		CL	0.33	0.16	0.67	0.01	0.56	0.28	1.17	0.02
	AdaBoost	Mean	8.40	54.05	28.88	0.29	7.41	53.55	26.88	0.30
		CL	0.31	0.15	0.54	0.01	0.60	0.30	1.10	0.03
	LDA	Mean	20.67	59.57	45.11	1.53	21.01	59.74	45.30	1.54
		CL	0.25	0.13	0.27	0.02	1.02	0.51	1.12	0.06
	KNN	Mean	1.49	50.68	11.87	0.13	1.66	50.77	11.12	0.13
		CL	0.17	0.08	0.78	0.01	0.41	0.21	1.80	0.02
	DT	Mean	16.33	56.38	39.62	3.57	16.25	56.33	39.38	3.60
		CL	0.49	0.25	0.60	0.05	0.89	0.45	1.11	0.12
	RF	Mean	1.68	50.79	12.71	0.09	1.63	50.78	11.30	0.08
		CL	0.18	0.09	0.71	0.01	0.35	0.18	1.65	0.01
	MLP	Mean	54.72	54.72	73.41	1.50	10.83	54.67	32.19	1.48
		CL	0.20	0.20	0.13	0.04	0.95	0.45	1.52	0.11
Balancing Methods	RO	Mean	93.43	89.00	88.87	15.43	61.57	73.01	68.56	15.55
		CL	0.36	0.78	0.79	1.27	5.14	2.14	5.63	1.84
	SMOTE	Mean	96.76	98.38	98.36	0.01	0.10	50.05	1.02	0.01
		CL	0.02	0.01	0.01	0.00	0.92	0.04	0.84	0.00

* CL = confidence level at 95%.

**Table 4:** Statistical tests performed on phase 1.

Performance	ANOVA		*t*-test			
	All Methods (Figure 6)		GA-CS-SVM vs. SVM (Figure 5)		GA-CS-SVM vs. RO (Figure 7)	
	F-Value	*p*-Value	T-Statistic	*p*-Value	T-Statistic	*p*-Value
Sensitivity	811.49	0.00	−117.41	0.00	4.65	0.00
AUC	628.32	0.00	−85.72	0.00	2.63	0.01
G-mean	486.17	0.00	−234.73	0.00	7.17	0.02
FPR	1302.70	0.00	−119.26	0.00	10.38	0.00

**Table 5:** Phase 2 results (training and LOOCV).

Models		Training				LOOCV			
		Sensitivity	AUC	G-Mean	FPR	Sensitivity	AUC	G-Mean	FPR
Proposed Approach	GS-SVM	80.00	79.29	79.28	21.43	75.56	75.06	75.06	25.43
Other Algorithms	SVM	53.33	69.10	67.27	15.14	59.26	72.49	71.27	14.29
	LR	54.81	73.55	71.12	7.71	44.44	66.79	62.94	10.86
	AdaBoost	54.07	72.61	70.20	8.86	42.22	64.54	60.56	13.14
	LDA	56.30	72.72	70.84	10.86	49.63	68.39	65.76	12.86
	KNN	51.11	73.41	69.94	4.29	35.56	63.21	56.84	9.14
	DT	100.00	100.00	100.00	0.00	45.19	63.45	60.76	18.3
	RF	100.00	100.00	100.00	0.00	45.93	69.39	65.39	7.10
	MLP	82.96	89.77	89.51	17.04	44.44	65.51	62.03	13.43
Balancing Methods	RO	100.00	95.14	95.05	9.71	94.29	86.00	85.60	22.29
	SMOTE	92.00	93.14	93.14	5.71	86.00	84.79	84.56	16.86

## Data Availability

These data were extracted from ChristianaCare Health Systems’ databases in Delaware, United States. It was de-identified for the purpose of this research. The data is not available for the public as it is owned by ChristianaCare.

## Supplementary Materials

The following supporting information can be downloaded at https://www.mdpi.com/article/10.3390/ijerph20010828/s1, Table S1: Models variables (features/risk factors for predicting phase 1 and phase 2), Figure S1. Database connection diagram.
