# Supplementary material for: An Integrated System of Multifaceted Machine Learning Models to Predict If and When Hospital-Acquired Pressure Injuries (Bedsores) Occur
Source: Int J Environ Res Public Health. 2023 Jan 1;20(1):828. doi: 10.3390/ijerph20010828 (PMC9820011; doi:10.3390/ijerph20010828)
Supplement: Supplementary file 1 [file ijerph-20-00828-s001.zip › ijerph-2062214-supplementary.pdf]

International Journal of
Environmental Research and Public Health

MDPI

Supplementary Table S1. Models variables (features/risk factors for predicting phase 1 and phase 2).

Feature Name	Type	Date Range	Features Selected by RFE for Phase 1	Features Selected by RFE for Phase 2
Age	Numerical	18-106 years		
Albumin*	Numerical	1.5-6 mmol/L	✓	
**Artificial Air Management**	Boolean	Yes, No	✓	✓
Blood Pressure Systolic*	Numerical	10-574 mmHg		
Blood Pressure Diastolic*	Numerical	6-912 mmHg	Average	
**Blood Urea Nitrogen (BUN)***	Numerical	2-293 mmol/L	✓	**Most Recent**
Body Mass Index (BMI)	Numerical	2.2-312.44		✓
**Comorbidity**	Boolean	Yes, No	✓	✓
**C-reactive Protein***	Numerical	5-604.1 mmol/L	✓	**First**
**Creatine Serum** *	Numerical	0.2-36.87 mmol/L	✓	**First**
Depression	Boolean	Yes, No		✓
Diabetes	Boolean	Yes, No		✓
Emergency Department Length-of-Stay	Numerical	0-353 days		✓
Face Mask	Boolean	Yes, No		
**Feeding Tube**	Boolean	Yes, No	✓	✓
Glasgow Coma Score*	Ordinal	3-15	✓	
Hemoglobin*	Numerical	3.7-18.2 g/dl	First and Most Recent	
High Mean Arterial Pressure (MAP)	Boolean	Yes, No	✓	
**ICU During Encounter**	Boolean	Yes, No	✓	✓
Lactate*	Boolean	3-19.5 mmol/L	First and Average	
Nasal Cannula	Boolean	Yes, No	✓	
Non-invasive Ventilation	Boolean	Yes, No		
**Number of Surgeries**	Numerical	0-12 surgeries	✓	✓
Opioids	Boolean	Yes, No	✓	
Palliative Orders	Boolean	Yes, No		
Patient Refusal to Change Position	Boolean	Yes, No		✓
Pharyngeal	Boolean	Yes, No		
Pressure Injury on Admission	Boolean	Yes, No	✓	
**Prior Year Inpatient Visit Counter**	Numerical	0-27 Visits	✓	✓
Pulse Oximetry*	Numerical	0-989 Pulse	Most Recent	

Renal Failure	Boolean	Yes, No	✓	
Room Air	Boolean	Yes, No	✓	
Sepsis Diagnosis	Boolean	Yes, No	✓	
**Sex**	Categorical	Male, Female	✓	✓
Skin Abnormality on Admission	Boolean	Yes, No		
Sodium*	Numerical	102-190 mmol/L		First and Most Recent
**Steroid History**	Boolean	Yes, No	✓	✓
Steroid Use	Boolean	Yes, No		✓
Stimuli Anesthesia	Boolean	Yes, No	✓	
Stimuli Paralytics	Boolean	Yes, No		
Stimuli Sedation	Boolean	Yes, No	✓	
Stimuli Tracheostomy	Boolean	Yes, No	✓	
Stroke History	Boolean	Yes, No		
Body Temperature*	Numerical	6.7-99.9 Fahrenheit		
Transitional During Encounter	Boolean	Yes, No		
Vasopressor	Boolean	Yes, No	✓	
Weight Loss	Boolean	Yes, No		
Ventilator	Boolean	Yes, No	✓	
Number of Pressure Injuries at Admission	Numerical	0-27 injuries	✓	
Overall Braden Score*	Categorical	Risky, Non-risky	✓	
**Activity Status***	Ordinal	Bedfast, Chairfast, Walks Occasionally, Walks Frequently	✓	✓
**Friction and Shear Status***	Ordinal	Problem, Potential Problem No, Apparent Problem	✓	✓
**Mobility Status***	Ordinal	Completely Immobile, Very Limited, Slightly Limited, No Limitations	✓	✓
**Nutrition Status***	Ordinal	Very Poor, Probably Inadequate, Adequate, Excellent	✓	**First and Average**
**Sensory Perception Status***	Ordinal	Completely Limited, Very Limited,	✓	✓

		Slightly Limited, No Impairment		
**Skin Moisture Status***	Ordinal	Constantly Moist, Often Moist, Often Moist, Rarely Moist	✓	✓
Race	Ordinal	American Indian or Alaska Native, Asian, Black or African American, Declined, Multiracial, Native Hawaiian or Other Pacific Islander, Other Race, Unknown, White		✓
Ethnic Group	Categorical	Hispanic or Latino, Not Hispanic or Latino, Declined, Unknown		
**Count of Glasgow Coma Score (GCS) Comments**	Numerical	143-26622 Comments	✓	✓
**American Society of Anesthesiologists (ASA) Score**	Ordinal	1-6	✓	✓

* Three factors were taken: First, Average, and Most Recent.

Bold: common features between Phase 1 and Phase 2.

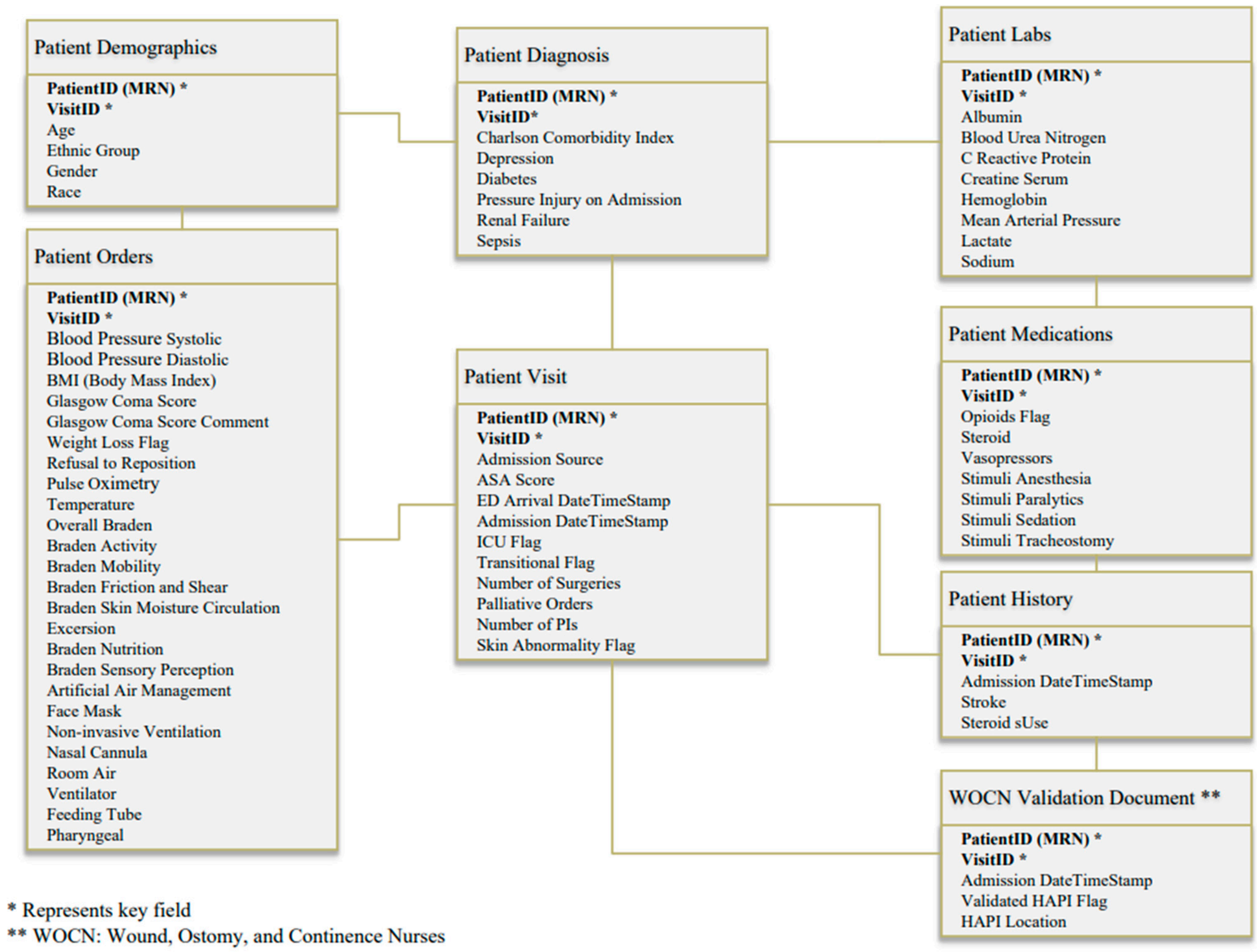

Supplementary Figure S1. Database connection diagram.
